# Longitudinal Surface-Based Morphometry Reveals Potential MRI Biomarkers Correlated with Multidimensional Brain Health Measures

**DOI:** 10.21203/rs.3.rs-8088216/v1

**Published:** 2025-11-19

**Authors:** Robyn A. Honea, Ankit Patel, William Guiler, Mark D’Esposito, Sandra Bond Chapman, Lori G. Cook, Jeffrey S. Spence

**Affiliations:** University of Kansas Alzheimer’s Disease Research Center; University of Kansas Alzheimer’s Disease Research Center; University of Kansas Alzheimer’s Disease Research Center; University of California, Berkeley; The University of Texas at Dallas; The University of Texas at Dallas; The University of Texas at Dallas

## Abstract

Characterizing longitudinal brain changes linked to brain health requires identifying structural and neuroanatomical biomarkers. This study aimed to discover MRI-based biomarkers for the Brain Health Index (BHI), a multidimensional measure of cognition, emotional well-being, and social connectedness, and assess their feasibility as indicators of brain health improvement. We investigated neuroanatomical correlates of BHI using longitudinal surface-based morphometry in 210 healthy adults (aged 20–70). We hypothesized that baseline brain morphology would predict BHI performance and that longitudinal BHI changes would correlate with morphometric changes. Participants underwent up to five MRI scans over four years. Surface-based analyses quantified changes in cortical volume, thickness, sulcal depth (SD), gyrification index (GI), and fractal dimension (FD). Vertex-wise regressions tested associations between baseline BHI and morphometric changes, and between longitudinal BHI changes and morphometric features, controlling for age, sex, education, and scan interval. In males, baseline BHI predicted increased FD in the left inferior parietal cortex. In the whole group, BHI gains correlated with increased GI in the left insula and posterior cingulate. In females, BHI improvements were linked to greater SD in the left precuneus, middle temporal, and right cingulate gyri. A sex-specific interaction showed BHI gains in males associated with increased GI in the left insula and temporal cortices. These findings highlight FD, GI, and SD as potential MRI biomarkers for tracking changes in brain health, revealing sex-specific neuroanatomical associations with BHI improvements in regions such as the insula and precuneus.

## 1. Introduction

The concept of brain health has emerged as a critical framework for understanding cognitive functioning, emotional well-being, and overall neural resilience throughout the lifespan. Most studies quantifying the relationship between measures of brain health and brain morphology are cross-sectional, limiting their generalizability. Chapman et al. have created a multi-domain indices of brain health that encompasses cognitive and social factors contributing to the whole human experience(Chapman et al., 2021). Unlike traditional approaches that focus narrowly on pathology or specific cognitive domains, the Brain Health Index (BHI) represents a holistic, multidimensional measure encompassing cognitive abilities, emotional well-being, social interactions and daily functioning(Chapman et al., 2021). This integrated measure offers significant advantages over conventional single-domain assessments by acknowledging the interdependent nature of neural systems that support multiple aspects of human functioning.

Hemodynamic, connectivity, and cerebrovascular metrics have been derived from the Brain Health Project data in relation to the factor structure of the Brain Health Index (BHI)(Spence et al., 2024). Individuals enrolled in the Brain Health Project engaged in online training and coaching sessions, and a pilot study of individuals aged 18–87 enrolled in this study showed age and sex independent gains in the data-driven index of brain health (BHI) (Chapman et al., 2021). Gains in the BHI have been recently associated with specific changes in the patterns of hemodynamic response (Spence et al., 2024), supported by studies in the literature showing correlations between both brain structure and function changes after cognitive training protocols (Fang et al., 2024; Haier et al., 2009; Zhang et al., 2025). However, it is still unclear whether changes in the BHI are associated with underlying changes in the brain’s neuroanatomy, as this has not yet been tested.

Surface-based neuroimaging analyses provide powerful tools for detecting subtle structural changes associated with improved brain health. While volumetric measures have predominantly been the focus in neuroimaging-based brain health intervention research, metrics such as cortical thickness, sulcal depth, and gyrification offer more nuanced insights into the morphological features that may reflect morphometric adaptations and optimization of neural circuits. These surface-based metrics can capture aspects of cortical organization that are more directly linked to functional specialization and cognitive abilities than gross volumetric measures alone (Marshall et al., 2025; Moodie et al., 2024). Surface-based morphometry (SBM) allows for vertex-based mapping that provides comprehensive information on the macrostructural relationships between cognition and brain health (Yun et al., 2013). For instance, one measure called local fractal dimension (FD) measures use spherical harmonic reconstructions that provide detailed information about the complexity of cortical folding (Yotter et al., 2011a; Yotter et al., 2011b) and may capture relationships with BHI that have not yet been identified. Sulcal depth (SD) is another surface-based measure defined as the linear distance between the central cortical surface, positioned midway between the gray matter-white matter boundary and the gray matter-cerebrospinal fluid boundary, and the corresponding point on the outer cortical hull (Dahnke et al., 2013). Sulcal measures are not dependent on the accurate identification of gray and white borders and are more sensitive to the folding of the cerebral surface(Lamont et al., 2014; Mangin et al., 2004). The gyrification index (GI), one of the most established and standardized metrics, is calculated as the ratio between the length of the inner contour (tracing the sulcal folds) and the outer contour, effectively representing a virtual hull that encompasses the brain’s surface (Zilles et al., 1988). Cortical thickness measures quantify the structural integrity of the brain’s gray matter, reflecting neurodevelopmental, degenerative, or adaptive changes in neuronal density, myelination and synaptic pruning (Fischl and Dale, 2000). Cortical thickness measurements have been successfully used in intervention trials to detect neuroplastic improvements, such as exercise-induced cortical normalization in pediatric brain tumor survivors (Timmons et al., 2018) and lifestyle interventions increasing frontotemporal thickness in older adults (Moon et al., 2022). We previously have used surface-based characterizations to quantify relationships between aging, genes, and disease (Honea et al., 2023; Honea et al., 2024).

The goal of this study was to identify potential structural MRI biomarkers for the BHI measure, and to assess the feasibility of these imaging metrics as biomarkers for brain health improvement. Thus, we characterized brain volume, thickness, fractal dimension, gyrification index, and sulcal depth in all subjects from their baseline to last scan (totaling 380 neuroimages). Morphological features of the brain underlie a range of psychological and cognitive processes. We hypothesized that features of brain morphology would predict longitudinal performance on the BHI. We also hypothesized that longitudinal change in BHI would correlate with longitudinal change in neurobiological features. We also tested for sex differences associated with these BHI-related changes in brain morphometry. Importantly, this exploratory analysis examines associations in a cohort with access to optional online brain health training, rather than testing a controlled intervention.

## 2. Materials and Methods

### 2.1. Participants

Prior to exclusions, 210 participants with at least two timepoints of neuroimaging data were selected from a larger cohort of participants enrolled in an ongoing study investigating relationships between indices of brain health described in previous work(Chapman et al., 2021). Recruitment for this study has been described elsewhere (Spence et al., 2024), but briefly participants completed an online screening prior to enrollment, were required to 1) be at least 18 year of age, 2) have reliable internet access on a computer, smartphone, or tablet, and 3) speak English proficiently. Those who completed screening were disqualified for ineligibility if they had 1) MRI contraindicators (e.g. claustrophobia, implanted devices, etc.), 2) had been diagnosed with any neurologic or psychiatric disorder, 3) had any history of traumatic brain injury, or 4) reasonably foresaw difficulties in keeping scanning session appointments.

All participants were informed about the study protocol and provided informed written consent prior to scanning. Although participants were not compensated financially for their participation, they were offered gift card incentives for completing online training milestones. Experimental procedures met all applicable standards and were approved by The University of Texas at Dallas (UTD) Institutional Review Board.

### 2.2. Indices of Brain Health

Brain health was assessed using the BrainHealth Index, a multi-dimensional composite metric derived from an online battery of cognitive assessments and self-report questionnaires evaluating cognitive, well-being, social, and daily life domains^1^. The Index is a validated tool that offers a unique neurobehavioral approach to assess brain health optimization rather than simply documenting losses to detect brain disease or injury or comparative performance against a set of norms typical of most traditional cognitive assessments. In contrast, the BrainHealth Index provides a sensitive and comprehensive tool for capturing holistic and multidimensional gains across the adult lifespan as well as losses over time, highlighting its utility in evaluating effectiveness of various intervention protocols across more than single or limited domains of cognitive or behavioral. The BrainHealth Index was designed to characterize dynamic change over time that could be validated by and linked to corresponding changes in brain structure and function.

**Figure 1:**
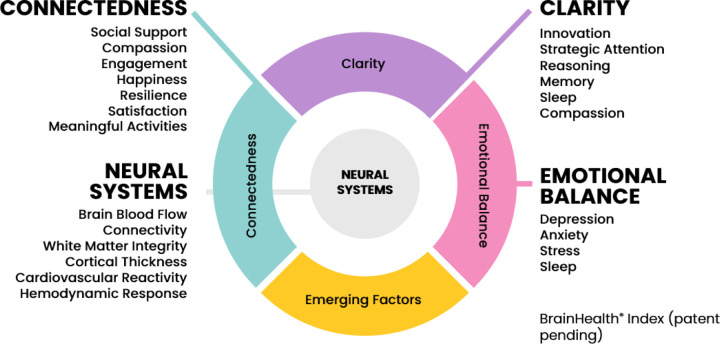
Brain Health Index Assessment Measures

In a pilot study of 144 participants without neuroimaging data, Chapman et al. used factor analysis to derive three constructs of brain health based on changes in the BrainHealth Index assessment measures listed in Fig. 1^1^. These three constructs were Connectedness (Factor 1), reflecting social and purpose-related measures (e.g., social support, engagement, compassion, happiness, resilience, satisfaction, and meaningful activities); Emotional Balance (Factor 2), based on assessments of mood (e.g., depression, stress, anxiety, happiness); and Clarity (Factor 3), encompassing largely cognitive metrics (e.g., strategic attention, memory, innovation, synthesis, generative fluency of abstract interpretations), along with metrics of sleep, compassion, and outlook (see Tables 1 and 4 in Chapman et al., 2021 for specific metrics and factor loadings). As described earlier, these three factors were later validated from a prospective neuroimaging study cohort using neural metrics – specifically, predictive hemodynamic response functions^2^. The BHI has also recently demonstrated utility in detecting gains associated with other interventions, such as menopausal hormone therapy(Fratantoni et al., 2025) and other cognitive training protocols(Young et al., 2025).

### 2.3. Online Brain Health Training

Upon completion of the baseline BrainHealth Index assessment, participants received their scores online and were instructed to schedule with a coach if they wanted more information. They then had access to online brain health training tools through the BrainHealth Platform (available via web and mobile app). Training was administered largely via micro-learning video modules available in small daily segments, ranging in time from 2–7 minutes. Initial training modules were based on the Strategic Memory Advanced Reasoning Tactics (SMART), a metacognitive strategy-based executive function training protocol, described elsewhere^1^. Later training modules instructed participants to apply the SMART principles and intentionally deploy tactical thinking strategies to other life applications, such as stress management, sleep, social relationships, health choices, and more. Note that we all use our brains throughout the day. SMART provides guidelines for how to deploy its use more intentionally to think deeply, innovate frequently through possibility thinking versus single solution approaches, focus, and single-task, and to stop toxic brain habits, such as constant distractions, information overload, and multitasking.

In addition to the online modules, participants were encouraged to access and utilize an online brain healthy habits in their everyday life and routines using an integrated brain-healthy habit-building tool. That is, they were guided to select various habits and practices they wanted to build and track overtime. Participants also had access to a curated selection of brain health-related informational resources, including online articles and recorded talks that provided the ‘why” with vetted neuroscience behind the habits. On a quarterly basis (every 3 months), participants could schedule an individual coaching session with a brain health coach which entailed a 20-minute videoconference call through the BrainHealth Platform. In addition, they could select to join a 45-minute group coaching session, offered every month on Zoom. Coaching. These sessions were optional and were meant to provide opportunities to learn from how others were utilizing the SMART executive function strategies and to support better understanding of the online assessment results and gain tips to guide goal-setting and personalized application of the brain health strategies and practices in daily life routines.

### 2.4. Scanning sessions

Imaging data were all collected at the Sammons BrainHealth Imaging Center at the UTD Center for BrainHealth (CBH). Data were collected on a Siemens Prisma 3 Tesla MRI scanner (Siemens Healthcare, Erlangen, Germany), equipped with a 32-channel head coil. Participants were imaged during up to 5 sessions at the time of this analysis, once at their baseline visit, then again at approximately 6 months, 18 months, 24 months, 30 months, and 42 months. The last available scan for each individual was chosen as their follow-up scan. Each participant was scanned for approximately 50 minutes using multiple sequences, including a T1-weighted magnetization-prepared rapid-acquisition gradient-echo (MPRAGE) used for structural analyses that was 192 sagittal slices (no gap) each containing a 256 × 256 matrix of 1mm^3^ isovoxels (flip angle = 8°, echo time = 1.12ms, repetition time = 2400ms).

### 2.5. Structural Data Preprocessing

Surface reconstruction was accomplished using the Computational Anatomical Toolbox (CAT12) (http://www.neuro.uni-jena.de/cat) run within the Statistical Parametric Mapping (SPM12) package (http://www.fil.ucl.ac.uk/spm/software/spm12/) in the Matlab environment (R2023b; https://www.mathworks.com). Patients were only included if their IQR value from the CAT12 homogeneity test was greater than or equal to 80. 25 participants were excluded due to scanning artifact or poor quality post-processing, with another 5 individuals who did not have adequate education data. We used custom SPM12 (CAT12) cross-sectional and longitudinal pipelines (Honea et al., 2022; Honea et al., 2016) and processed all data using custom protocols for multiple time points with T1 MPRAGE data.

### 2.6. Longitudinal Analysis

For the longitudinal pipeline, baseline and follow-up images were co-registered for each individual and then realigned across the entire sample for the last timepoint individuals had a scan. The preprocessing pipeline further including bias correction, image segmentation (i.e. into cerebrospinal fluid (TICV), white matter (WM), gray matter (GM), and white matter hyperintensity (WMH)), transformation into MNI space and DARTEL normalization. Data were smoothed with a 15-mm (for CT) or a 25-mm (for FD, GI, SD) FWHM Gaussian kernel, and delta images for each individuals first and last scan at the time of this study were created via CAT 12’s cat_stat_diff function (i.e. “follow-up” minus “baseline”). For all analyses, voxels are reported with reference to the MNI standard space within SPM12. To avoid possible edge effects at the border between GM and WM and to include only relatively homogeneous voxels, we used an absolute threshold masking of 0.10 for each analysis.

### 2.7. Neuroimaging statistics

Statistics were done in imaging space across all voxels. We investigated whole-brain longitudinal changes of different volumes and surface-based measures within imaging space using a regression model, covarying for age, sex, education and months between scans to capture various aspects of brain morphology associated with a)baseline BHI score and b)change in BHI score from the first to last visit. We followed all analyses with sex-stratified analyses, due to known morphometric differences between men and women, looking at men and women separately, as well as an interaction analysis of sex by change in BHI. Family-wise error (FWE) correction was applied to the entire brain, and we considered a corrected p < .05 as significant. We also ran non-parametric statistical analyses using the threshold-free cluster enhancement method (TFCE) (Smith and Nichols, 2009), which allows for cluster-based inference without the need to pre-specify arbitrary thresholds. This implementation in the TFCE toolbox for CAT12 performs parametric permutation tests, thus avoiding problems inherent to parametric statistics (Eklund et al., 2016), and has been recommended in similar SBM-based whole-brain analyses (Bachmann et al., 2023). Anatomical labeling from the Wakeforest Pickatlas AAL atlas was used to identify peak coordinate regions in VBM and SBM. The Desikan-Killiany (Desikan et al., 2006) atlas was used for SBM (and AAL for VBM) to extract mean regional values from the processed images in significant regions after voxel-wise analysis.

### 2.8. Statistical Analyses

SPSS 23.0 (IBM Corp., Armonk, NY) was used for the statistical analyses performed outside of imaging space. Continuous demographic, BHI, and global volumetric imaging variables were presented for the overall group as well as compared between men and women for the descriptive statistics. A chi-square analysis was used to compare categorical demographic variables between groups.

## 3. Results

We report baseline and demographic and participant characteristics in Table 1, separated by sex as we did a secondary analysis separating sex. Our sample consisted of 190 healthy individuals aged 20–70 (mean age 43.75), consisting of 94 women and 96 men. Previous analyses in this sample have confirmed that age, sex and education levels are not an underlying mediating factor influencing change in the BHI(Ajith et al., 2024; Chapman et al., 2021; Spence et al., 2024). Note that we systematically corrected for sex (and age) in the following analyses, as there are known differences in brain morphometry across sex and age. In our sample men had significantly larger (p < .001) TICV (mean = 1588 (SD = 120.8)) than women ((mean = 1405 (SD = 96.6)), as well as GM normalized to TICV (p = .02). Mean BHI in the whole sample was 697.46 (standard deviation (SD) = 74), with an average change of 33.3 (SD = 70) points over an average of 14.1 months between scans (SD = 8.5). Scan quality IQR rating was 86.3 (SD = 1.3)

### 3.1. Longitudinal changes in cortical complexity

**Figure 2:**
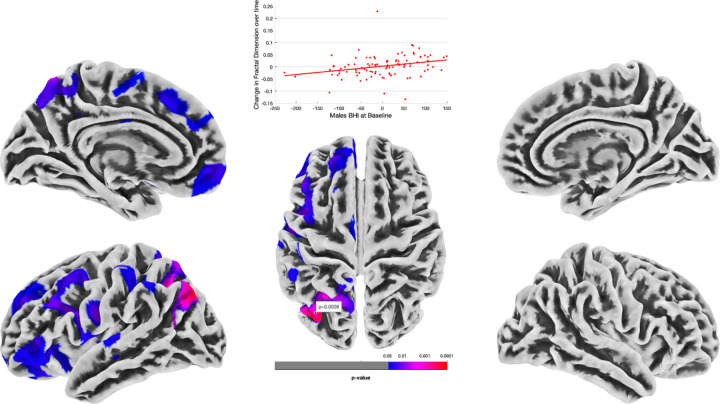
Clusters (in blue-pink) showing significant relationship between baseline BHI score and increased fractal dimension over time in males. Left inferior parietal cortex cluster plotted at vertex for visual purposes against baseline BHI (from TFCE FWE corrected analysis). LH (RH): left (right) hemisphere

Our first series of multiple regression analyses in-vivo characterized the predictive relationship between baseline BHI score and brain morphometry change over time. There were no significant relationships between baseline BHI score and GMV, CT, GI or SD either in the whole group or in men or women separately. However, in the surface-based morphometry analysis of baseline BHI and change in Fractal Dimension over time in men, there was a significant relationship in the left inferior parietal cortex (117 vertexes, TFCE value of 3500.91, combined peak/cluster level FWE-corrected TFCE p = .049, MNI coordinates at −32, −76, 43) (Fig. 2).

**Figure 3:**
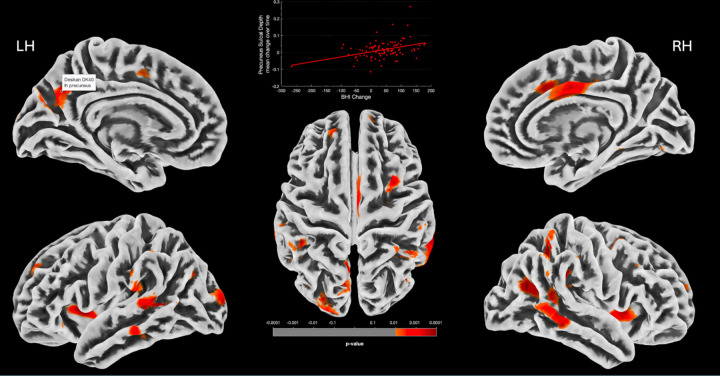
Clusters (in yellow-red) showing significantly increased sulcal depth correlated with increase in BHI score over time in females. Right cingulum cluster plotted at vertex for visual purposes against change in BHI (from Cluster-level FWE corrected analysis). LH (RH): left (right) hemisphere

**Figure 4:**
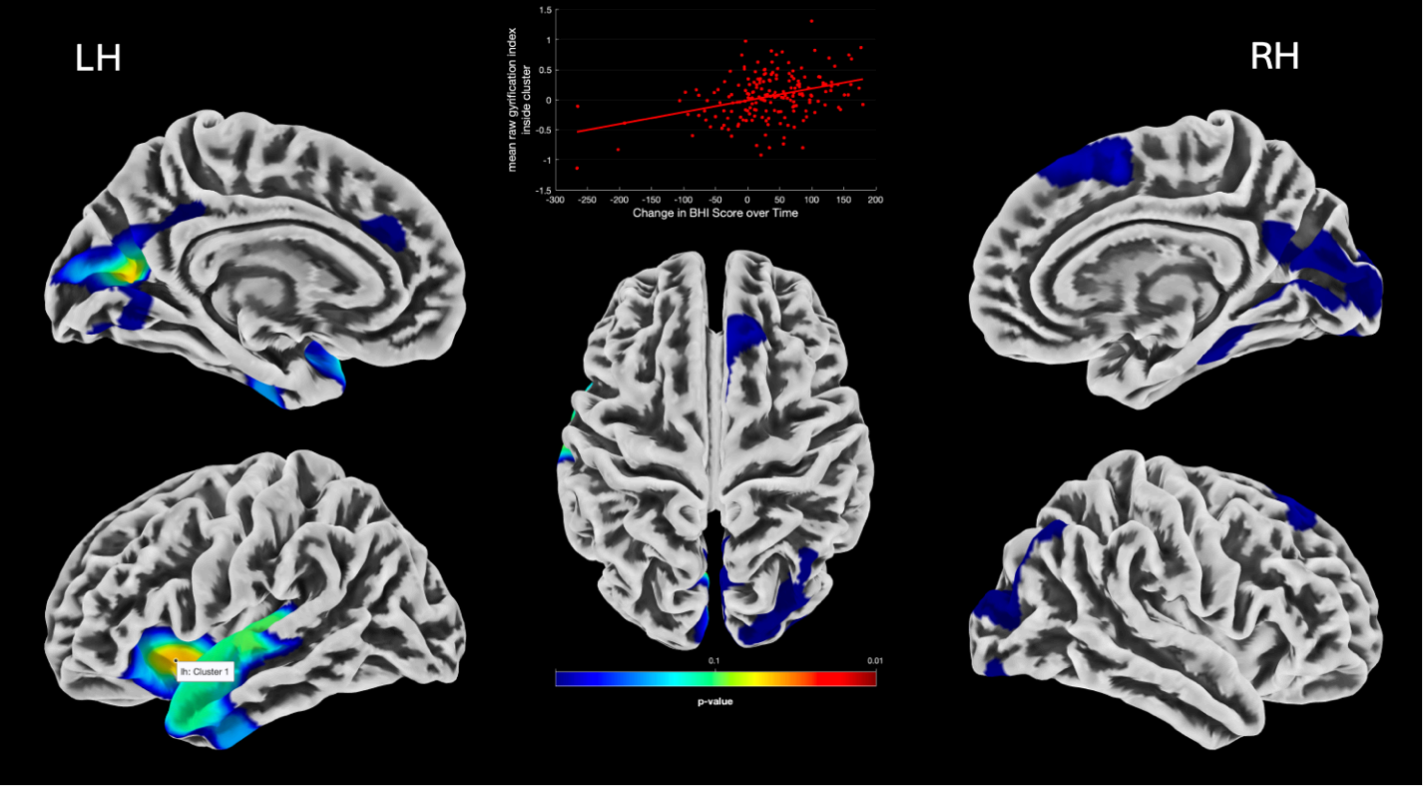
Clusters (in yellow) showing significantly increased gyrification index correlated with increase in BHI score over time in the whole group. Left insular cluster plotted at vertex for visual purposes against change in BHI (from TFCE FWE-corrected analysis). LH (RH): left (right) hemisphere

**Figure 5:**
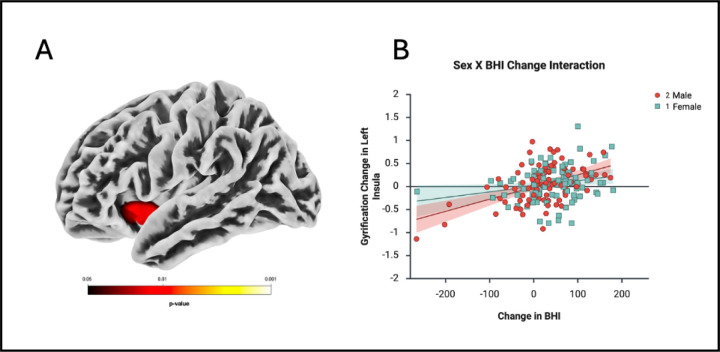
**A.** Cluster (in red) showing a sex-specific interaction of a significantly increased gyrification index correlated with an increase in BHI score. **B.** Left insular cluster plotted at vertex for visual purposes against change in BHI (from TFCE FWE-corrected analysis). LH (RH): left (right) hemisphere

Our second series of regression analysis investigated longitudinal relationships between brain morphometry change over time and change in BHI scores, with results detailed in Table 2 and Table 3. In the voxel-based analysis of gray matter volume (GMV) and surface-based analysis of cortical thickness (CT), and fractal dimension (FD) changes over time associated with BHI change; there were no regions reaching statistical significance in the whole group, in males or in females. In the surface-based analysis of sulcal depth, there was a significant relationship between increased sulcal depth and increased BHI over time, specifically in women, in the left precuneus, left middle temporal gyrus, and right cingulate gyrus at a cluster-level FWE corrected significance (Table 2, Fig. 3). In the surface-based analysis of gyrification change there was a significant relationship between increase in gyrification index and BHI change in the whole group in the left insula and left posterior cingulate at FWE-corrected TFCE p < .05 (Table 3, Fig. 4). When looking at males and females separately, the same cluster that was associated with BHI change and GI change in the whole group in the left insula was still significantly associated with GI change in men at both cluster-level FWE and FWE-corrected TFCE p < .05 (Fig. 5). Moreover, in the interaction model of sex by BHI change there was a significant sex interaction on gyrification change at FWE-corrected TFCE p < .05 at a cluster encompassing the left insula, left middle temporal gyrus and left superior temporal gyrus (Table 3)

## 4. Discussion

The study aimed to identify structural MRI biomarkers for the BHI measure and to evaluate their potential as indicators of improved brain health. These findings provide evidence of associations between changes in cortical morphometry and enhanced brain health over time. Gyrification and sulcal depth increases were correlated with enhanced brain health in this cohort, suggesting possible structural adaptations across the adult age span (ages 18 onwards) among participants with access to cognitive training resources in the Brain Health program. There was also a sex-specific interaction between change in BHI and change in brain gyrification, which manifested as BHI improvements in men correlated with increased gyrification in the left insula and left temporal cortices. These observed structural changes, alongside baseline predictors, serve as potential MRI biomarkers associated with cognitive enhancement, supporting the feasibility of cortical complexity metrics in tracking changes related to the impact of BHI.

We frame these findings as reflecting two types of structural metrics: trait-like predictors and state-like changes. Baseline brain structure, as a trait, predicted BHI improvements, suggesting its reliability as a biomarker for brain health. In males, higher baseline BHI scores were associated with increased FD over time in the inferior parietal sulcus over time, suggesting that cortical complexity at baseline may be linked to intervention response, akin to brain modularity’s predictive role (Baniqued et al., 2019; Gallen et al., 2016). Fractal dimension is a morphometric measure that has been increasingly used to study the changes of brain shape complexity in aging and neurodegenerative disease (Honea et al., 2023; Ziukelis et al., 2022). Preserved fractal dimension has been associated with healthy aging, whereas decreases in fractal dimension in the parietal cortices have been linked to reduced cognition and Alzheimer’s disease. A study in patients with mild cognitive impairment and small vessel disease showed that a decrease in white matter fractal dimension, indicating reduced structural complexity, was associated with worsening cognitive performance (Pantoni et al., 2019). Similarly, Mustafa et al. (2012) found that healthy individuals with reduced white matter fractal dimension had lower intelligence scores and greater age-related cognitive decline (Mustafa et al., 2012). In contrast, our study’s finding of increased fractal dimension in men with higher baseline BHI scores, specifically in the inferior parietal sulcus, suggests structural optimization associated with enhanced brain health. This region’s role in cognitive processes supports its biomarker potential for identifying individuals primed for BHI improvement (Capizzi et al., 2023; Sebastian et al., 2013; Tabassi Mofrad and Schiller, 2025).

Structural metrics as a state reflect changes observed over time, such as increased GI and SD. We found increased gyrification index in men and women in the left insula and left posterior cingulate gyrus associated with increases in the BHI over time. Gyrification patterns serve as sensitive structural neuroimaging biomarker for brain disorders and also brain health; alterations in GI have been specifically observed in various neurological disorders (Gaser et al., 2006; Libero et al., 2019; Matsuda and Ohi, 2018; Sterling et al., 2016) and have been linked to functional changes in the brain (Kelly et al., 2013; Schaer et al., 2013). Increased gyrification in the insula was associated with improved BHI scores in our study, particularly in men as seen in our interaction analysis. In contrast, a recent study of individuals with peripheral nerve degeneration showed decreased gyrification in the insula, with a greater reduction in gyrification linked to more severe skin nerve degeneration (Lin et al., 2025). The insula exhibits unique sulcal morphology (shallower sulci, lower fractal dimension) compared to other lobes, reflecting its distinct growth trajectory (Mallela et al., 2023). The structural integrity in the insula, as evidenced by the increased gyrification in our study, may reflect adaptations possibly underlying the complex network activity that possibly contributed to enhanced BHI scores. While the mechanisms underlying these state changes are unclear, they may involve synaptic remodeling, dendritic growth, or neurovascular coupling, as suggested in cognitive training studies (Chapman et al., 2015; Engvig et al., 2010). The insula’s role as a hub connecting various brain networks, including the salience network and higher-order cognition, has been documented in prior research (Namkung et al., 2018). It serves as a hub connecting various brain networks, including the salience network, which facilitates access to cognitive resources during the detection of salient events. A recent 6-month multi-domain lifestyle intervention study (SUPERBRAIN) found significantly increased regional cortical thickness in the insula after intervention (Moon et al., 2022). Similar to our study, they did not find gray matter volume changes as a whole alongside increased thickness measurements (Moon et al., 2022). These data support previous work by our group which found a functional relationship of neurophysiologic metrics, specifically change in HRF amplitude (Spence et al., 2024), with the change in BHI. This brain-behavior relationship helps argue that the possible mechanism for changes in BOLD hemodynamics reported across time associated with this measure may be related to subtle but important changes in brain morphometry. Prior studies have shown that functional brain changes precede structural ones post- SMART training (Chapman and Mudar, 2014), suggesting neural efficiency drives morphological adaptations (Burzynska et al., 2013; Rypma et al., 2006).

In women, we found increased sulcal depth associated with improved BHI scores in the precuneus, cingulate, and middle temporal gyrus. Prior studies have associated the precuneus, a highly connected and metabolically active brain region, with cognitive functions such as episodic memory, working memory, and self-referential processing (Dominguez et al., 2021; Leech and Sharp, 2014). Deeper sulci may reflect optimized white matter organization(Willbrand et al., 2024) or preserved gyral volumes(Jin et al., 2018), possibly underlying BHI’s cognitive and emotional domains. Research has demonstrated that the structural integrity of the precuneus is associated with preserved cognition in successful aging. Studies examining older adults with exceptional cognitive abilities compared to their cognitively typical peers have consistently identified the precuneus as a region showing greater cortical thickness(Dominguez et al., 2021). Studies have demonstrated that in Alzheimer’s disease, for instance, the sulci widen and become more shallow. Conversely, our findings suggest that increased sulcal depth may represent a positive structural change underlying network changes associated with enhanced brain health. To our knowledge, ours is the first study to show state-related changes with the metric of sulcal depth in the middle temporal cortex associated with a cognitive training intervention. However, a previous study has shown increased memory retrieval activity in the middle temporal cortex after memory training in healthy older adults (Belleville et al., 2011). Deeper sulci in this region may underly functional mechanisms involved in neural processing for language and memory functions, which are components of the cognitive domain assessed in the BHI. This finding aligns with the multidimensional nature of the BHI, which incorporates cognitive, including memory and complex information processing of language, social connectedness, and emotional well-being metrics. Increased sulcal depth in the cingulate gyrus may underly the integration of emotional and cognitive processes that contribute to overall brain health in women. For instance, functional MRI and EEG studies have shown that the cingulate may support emotional regulation, cognitive regulation through attention, and performance monitoring function(Bush et al., 1999; Bush et al., 2000; Bush et al., 2022). There are also recent studies showing that sulcal pits predict cognitive skill such as verbal IQ and that the depth of sulcal pits are correlated with different aspects of cognition(Natu et al., 2021). Preserved sulcal depth in older adults is linked to better maintenance of local gyral volumes, suggesting a protective effect against age-related decline(Jin et al., 2018). Our observed increases in sulcal depth over time in this region align with findings from Spence et al. (Spence et al., 2024), who demonstrated that neural network models could accurately predict cognitive index trajectories from hemodynamic response function parameters. Their work emphasized that neuroimaging measures can track cognitive indices in healthy states with the BHI, providing support for precision brain health approaches that map structural integrity to functional outcomes. Our findings extend this work by suggesting that for women, the structural optimization of the precuneus may represent a key neuroanatomical correlate of enhanced brain health.

Relationships between changes of cortical complexity and external interventions are complex to investigate, however some research does exist. For instance, several studies, including a recent exercise study (Scharf et al., 2024), demonstrated that 12 weeks of physical exercise breaks with coordinative exercise training increased sulcal depth in the inferior parietal lobe, a region where we also saw increased sulcal depth. Lamont et al. (Lamont et al., 2014) argue that changes in brain structure due to regular physical activity can be detected earlier in sulcal measures compared to more typical volumetric measures. Tasks that draw on visuomotor abilities in particular have been known to activate the intraparietal sulcus (Capizzi et al., 2023). Moreover, the SUPERBRAIN study showed lifestyle intervention impacted cortical thickness after only 6 months (Moon et al., 2022). Another multi-domain cognitive training study also showed significant effects of their intervention on the left supramarginal gyrus thickness associated with cognitive change scores (Jiang et al., 2016). Working memory training over 8 weeks in middle-aged individuals resulted in increased cortical gyrification in bilateral parietal regions as well as changes in surface area in the occipital cortex (Wu et al., 2021). A recent study of 8 weeks of standardized computerized working memory training found changes in cortical thickness, fractal dimension, morphometric similarity network changes, and associated these network datasets with gene transcript expression (Zhang et al., 2025 CNS Neuroscience & Therapeutics).

It is our opinion that structural training-induced changes may reflect improvements in the brain processing networks at a neuroanatomical level, however these underlying mechanisms from the neurons, glia and axons have yet to be identified. Trachtenberg et al. (Trachtenberg et al., 2002) argued that neural activity changes involve an underlying spine and synapse turnover, as one explanation. Along this line, a change in size in the soma and nucleus of neurons, as well as glia and capillary dimensions have been shown to influence cortical morphology in animals (Muotri and Gage, 2006). Animal training studies using high resolution MRI and immunohistochemistry have shown MRI-derived volume changes after training correlated with axonal growth-associated protein-43 (GAP-43), indicating that the measured morphological changes are due to remodeling of neuronal processes and not due to neurogenesis (Lerch et al., 2011). Increases in DTI measures of FA in rats due to spatial learning has been linked to increase in oligodendrocytes forming the myelin sheaths over neurogenesis (Blumenfeld-Katir et al., 2011). Another source of training-induced changes in MRI metrics could be increase in density of capillaries, at least in the case of an animal exercise training model (Morland et al., 2017). Future studies should include plasma measurements on factors like BDNF and VEGF to get closer to the level of synaptic and vascular mechanisms, and combine fMRI, DTI, and vascular imaging with SBM to link state changes to function, and validate biomarkers in diverse cohorts.

Accumulating evidence indicates that men and women display distinct neuroanatomical responses to behavioral interventions that bolster brain health. Comparable divergence to our study emerges with physical exercise, as meta-analyses and longitudinal imaging show older women gain greater executive-function improvements and selective enlargement of hippocampal subfields and prefrontal cortex, whereas men exhibit different volumetric changes despite similar activity levels(Barha et al., 2017; Barha and Liu-Ambrose, 2020). Likewise, a six-week multidomain cognitive-training program in mild cognitive impairment produced stronger gains in immediate and delayed verbal memory and working memory for women than men, echoing functional-MRI evidence of differential network recruitment during task performance(Rahe et al., 2015). A study on long-term meditation practices showed sex-specific hippocampal expansion, left-lateralized in men and right-lateralized in women, which paralleled larger stress-reduction benefits reported by female practitioners(Luders et al., 2015). These studies underscore the importance of a sex-informed lens when evaluating structural MRI biomarkers as a state for cognitive enhancement and brain health, and provide mechanistic context for the sex-specific gyrification and sulcal-depth changes linked to BHI improvement in our cohort.

Improvements or decreases in BHI over time may stem from interventions (e.g., cognitive training, exercise, or social activities) or natural enhancements in these domains; however, there is a large amount of inter-individual variability that may not be captured in the measures making up BHI. Individuals in this study also varied in their number of visits and length between first and last brain scans, which we considered as a covariate to control for these differences; however, in doing so, we may have removed some variability important to understanding associated brain structure changes to BHI. Neuroanatomical biomarkers are limited to measuring structure, as opposed to network-level interactions in the brain, like modularity, which most likely underly the complex changes that occur during intervention-related plasticity in the brain(Gallen and D'Esposito, 2019). Future studies should combine structural and functional neuroimaging data to investigate network-level plasticity, especially network modularity, which examines network modules and connection types associated with plasticity in the brain (Gallen and D'Esposito, 2019). Baseline brain modularity may also be a key underlying measure of network architecture that explains why some individuals are able to improve over time and others do not.

In summary, our results indicate that there are morphometric biomarkers that may be useful for understanding measures brain health. Baseline surface-based measurements (FD), as a stable trait, predict BHI improvements, and GI/SD as a state, reveal subtle, regionally distinct changes over time that are significantly associated with longitudinal changes in the BHI. By identifying specific neuroanatomical correlates of brain health improvement, this study takes an important step towards precision brain health, where interventions can be tailored to individual neuroanatomical profiles to optimize cognitive, emotional, and social functioning throughout the lifespan. Our findings demonstrate the value of combining multidimensional behavioral brain health indices and advanced morphometric methods to identify biomarkers that reflect positive brain health trajectories. We propose that the structural changes may be achieved through activation of latent neuroplasticity capabilities that remain viable throughout adulthood. In the future, tailoring interventions to individual neuroanatomical profiles may serve to personalize and optimize cognitive, emotional, and social functioning throughout the lifespan.

## Figures and Tables

**Table 1:** Participant demographics

Mean (SD)	All n = 190	Female n = 94	Male n = 96	P-value (sex)
**Age**	43.75 (20–70~)	44.2 (12)	43.3 (12.2)	0.627
**BHI**	697.46 (74)	704.4 (72)	690.7 (74.9)	0.204
**BHI Change**	33.3 (70)	41.9 (67.9)	24.9 (71.3)	0.094
**Months between scans/BHI**	14.1 (8.5)	13.9 (7.7)	14.3 (9.2)	0.788
< Bachelors* (#)	24	10	14	0.604
Bachelors* (#)	84	46	38	0.604
>Bachelor's* (#)	82	38	44	0.604
**Average Neuroimaging IQR**	86.3 (1.3)	86.3 (1.2)	86.3 (1.3)	0.728
**TIV (mm3)**	1497.8 (142)	1405 (96.6)	1588 (120.8)	<.001
**Gray Matter Volume, TIV adjusted**	.462 (.02)	.466 (.02)	.457 (.02)	0.02
**White Matter Volume, TIV adjusted**	.351 (.01)	.349 (.02)	.353 (.02)	0.195
**White Matter Hyperintensity Volume (mm3)**	1.88 (2.06)	1.62 (.63)	2.15 (2.8)	0.076

Demographic, neuropsychological, and MRI characteristics of the individuals from the VBM and SBM analysis. Values are mean (SD (standard deviation)). TIV; Total Intracranial Volume, mm; millimeter, BHI; Brain Health Index, IQR; Image Quality Rating scale, n; number.

**Table 2:** Surface Based Morphometry Analysis; Sulcal Depth

Comparison	Size (vertices)	Peak t value	Cluster level p value (FWE) corrected	uncorrected p value	Coordinates (mm mm mm)	Brain Region
**Sulcal Depth**						
**BHI Change Pos (Females only)**	**234**	**3.61**	**0.001**	**<.001**	**−6 −61 40**	**L Precuneus**
		3.31		0.001	−11 −66 24	L Cuneus
		3.3		0.001	−5 −70 32	LPrecuneus
		3.25		0.001	−5 −63 34	L Precuneus
		3.25		0.001	−9 −68 30	L Precuneus
		2.75		0.004	−14 −62 20	L Cuneus
		2.72		0.004	−13 −68 34	L Precuneus
		2.59		0.006	−18 −68 28	L Superior Occipital Cortex
	**173**	**3.4**	**0.01**	**0.001**	**−50 −48 5**	**L Middle Temporal Gyrus**
		3.38		0.001	−55 −37 2	L Middle Temporal Gyrus
		3.38		0.001	−58 −41 5	L Superior Temporal Gyrus
		3.35		0.001	−61 −43 7	L Superior Temporal Gyrus
		3.33		0.001	−65 −38 6	L Superior Temporal Gyrus
		3.21		0.001	−56 −45 8	L Middle Temporal Gyrus
		3.12		0.001	−53 −42 5	L Middle Temporal Gyrus
		2.95		0.002	−52 −53 6	L Middle Temporal Gyrus
	**251**	**3.19**	**<.001**	**0.001**	**3 −5 36**	**R Middle Cingulate Gyrus**
		2.82		0.003	7 −20 41	R Middle Cingulate Gyrus
**Gyrification**						
**BHI Change Pos (Malesonly)**	**327**	**4.14**	**.022**	**<.001**	**−39 11 −7**	**L Insula**

Significant Results from Surface Based Morphometry analysis listed at a threshold of p < .05 FWE Cluster-level corrected, and TFCE Combined Cluster-peak level corrected, covariates included were age, education, and months between scans. primary peaks within cluster bolded in table. Coordinates listed are Montreal Neurological Institute. L; Left, R; Right.

**Table 3:** Surface Based Morphometry Analysis; Gyrification

Comparison	Size (vertices)	TFCE	Combined peakcluster- level (FEW Corrected for TFCE)	uncorrected p value	Coordinates (mm mm mm)	Brain Region
**Gyrification**						
**BHI Change Pos (everyone)TFCE**	**2410**	**3853.51**	**0.043**	**0.001**	**−38 11 −8**	**L Insula**
		3196.24	0.094	0.001	−61 −16 4	L Superior Temporal Gyrus
		3117.36	0.103	0.001	−61 −6 −1	L Superior Temporal Gyrus
		3085.39	0.107	0.001	−56 −0 −4	L Superior Temporal Gyrus
		3084.69	0.107	0.001	−54 2–7	L Superior Temporal Gyrus
		3050.98	0.112	0.001	−55 6–10	L Temporal Pole
		2659.15	0.176	0.002	−52 −11 −14	L Middle Temporal Gyrus
		1452.78	0.631	0.015	−53 −34 −1	L Middle Temporal Gyrus
		1251.79	0.754	0.018	−50 −22 −10	L Middle Temporal Gyrus
	**1295**	**3792.18**	**0.046**	**0.001**	**−11 −68 8**	**L Posterior Cingulate**
		626.41	1	0.049	−9 −102 1	L Cuneus
**BHI Change Pos**	**1323**	**5971.09**	**.007**	**<.001**	**−39 11 −7**	**L Insula**
**(Malesonly)**						
		3203.18	0.107	0.001	−59 1–8	L Middle Temporal Gyrus
		3162.59	0.111	0.001	−63 −12 0	L Superior Temporal Gyrus
		1601.49	0.529	0.006	−50 13–21	L Temporal Pole
		1418.75	0.633	0.008	−47 7–29	L Temporal Pole
**BHI Change Pos (everyone) × sex TFCE**	**1582**	**5544.58**	**0.009**	**<.001**	**−38 11 −8**	**L Insula**
		3140.35	0.108	0.001	−59 2–10	L Middle Temporal Gyrus
		3109.63	0.111	0.001	−61 −6 −1	L Superior Temporal Gyrus

Significant Results from Surface Based Morphometry analysis listed at a threshold of p < .05 TFCE FWE Combined Cluster-peak level corrected, covariates included were age, education, and months between scans. Primary peaks within the cluster are bolded in the table. Coordinates list Primary peaks within the cluster are bolded in the table.Left, R; Right;

## Data Availability

Data will be made available on request.

## Data Availability

Data will be made available on request.
